# Environment-dependent prey capture in the Atlantic mudskipper (*Periophthalmus barbarus*)

**DOI:** 10.1242/bio.019794

**Published:** 2016-10-07

**Authors:** K. B. Michel, P. Aerts, S. Van Wassenbergh

**Affiliations:** 1 Functional Morphology Laboratory, Department of Biology, University of Antwerp, Universiteitsplein 1, Antwerp 2610, Belgium; 2 Département d'Ecologie et de Gestion de la Biodiversité, UMR 7179 C.N.R.S/M.N.H.N., 57 rue Cuvier, Case Postale 55, Paris Cedex 05, 75231, France

**Keywords:** Mudskipper, *Periophthalmus*, Aquatic feeding, Terrestrial feeding, Comparison

## Abstract

Few vertebrates capture prey in both the aquatic and the terrestrial environment due to the conflicting biophysical demands of feeding in water versus air. The Atlantic mudskipper (*Periophthalmus barbarus*) is known to be proficient at feeding in the terrestrial environment and feeds predominately in this environment. Given the considerable forward flow of water observed during the mouth-opening phase to assist with feeding on land, the mudskipper must alter the function of its feeding system to feed successfully in water. Here, we quantify the aquatic prey-capture kinematics of the mudskipper and compare this with the previously described pattern of terrestrial feeding. Prior to feeding in the aquatic environment, the gill slits open, allowing water to be expelled through the gill slits. The opposite happens in terrestrial feeding during which the gill slits remain closed at this point. In water, the expansive movements of the head are larger, amounting to a larger volume increase and are initiated slightly later than in the terrestrial environment. This implies the generation of strong suction flows when feeding in water. Consequently, the kinematic patterns of the hydrodynamic tongue during terrestrial feeding and aquatic suction feeding are similar, except for the amplitude of the volume increase and the active closing of the gill slits early during the terrestrial feeding strike. The mudskipper thus exhibits the capacity to change the kinematics of its feeding apparatus to enable successful prey capture in two disparate environments.

## Abstract (precis)

**Summary:** A comparison of the kinematics of aquatic and terrestrial feeding in the mudskipper shows how the ancestral kinematic pattern of aquatic feeding is modified to enable the terrestrial capture of prey.

## INTRODUCTION

Capturing prey in both the aquatic and terrestrial environment presents numerous challenges because of the different physical demands on the feeding system to enable it to function in water as well as in air (e.g. Deban and Wake, 2000; Deban, 2003). Aquatic vertebrates generally use suction of water into the mouth, which is achieved by expanding the buccal and pharyngeal cavities during feeding (e.g. Muller and Osse, 1984; van Leeuwen and Muller, 1984; Lauder and Shaffer, 1985). Terrestrial vertebrates have evolved capture modes where direct contact with the prey by the jaws and/or tongue is used (e.g. Findeis and Bemis, 1990; Schwenk, 2000; Herrel et al., 2012). Animals that capture prey in both environments will generally have a feeding system that favours performance in one of the environments, or else they will have evolved a feeding system that fully compensates for the different biomechanical demands (Bramble, 1973; Stayton, 2011).

Among tetrapods, only a few species of salamanders and turtles have a truly amphibious feeding system. Recent studies have shown how terrestrial and aquatic turtles, and also salamanders, alter their feeding behaviour and kinematics to function in the medium they inhabit (Summers et al., 1998; Stayton, 2011; Heiss et al., 2013). Amphibious salamanders and turtles are capable of performing suction feeding in the aquatic environment. However, in the terrestrial environment, these turtles and salamanders increase the extent and duration of the movements from their aquatic feeding patterns and generally perform sub-optimally. More terrestrially acclimatized turtles and salamanders (turtles that are habitualised to the terrestrial environment and salamanders in their terrestrial mode) use an alternative feeding pattern on land, either by modifying their aquatic feeding pattern or by using morphological adaptations, such as the tongue (Stayton, 2011; Heiss et al., 2013). These studies show how the amphibious lower tetrapods manage to feed both in water and on land by using of a repertoire of movements of the head, the hyoid and oral jaws and/or by using specific morphological adaptations. But how do the feeding patterns of the amphibious vertebrates that have retained many of the characteristics of the cranial system of their aquatic ancestors, such as the amphibious fish, change between environments?

Mudskippers are known for their amphibious lifestyle on the intertidal mudflats in tropical river estuaries (Stebbins and Kalk, 1961). Some species of mudskipper have been reported to spend over 90% of their time on land (Gordon et al., 1969). They primarily seek their food out of the water and prey detection occurs chiefly by sight (Stebbins and Kalk, 1961). The feeding system of the mudskippers has been reported to function effectively in the terrestrial environment (Stebbins and Kalk, 1961; Sponder and Lauder, 1981). Based on recent studies, we have learned how the Atlantic mudskipper (*Periophthalmus barbarus*) pivots the head down, supported by their strong pectoral fins, to allow the oral jaws to be placed over their terrestrial prey (Michel et al., 2014). The terrestrial feeding is further aided by the use of water retained in the buccal cavity. The mudskipper first compresses the buccal cavity, forcing water forward towards its mouth. This water is subsequently sucked back by expansion of the buccal cavity (Michel et al., 2015). In doing this, the mudskipper often manages to transport its prey to the pharyngeal jaw region in a single gape cycle (Michel et al., 2015).

The considerable compression of the buccal cavity and the consequent anterior flow of water observed during the entire phase of mouth opening when feeding in the terrestrial environment (Michel et al., 2015), raises questions about the aquatic feeding ability of the mudskipper. This anterior pumping of water is in the opposite direction to the posterior flow of water generated during suction feeding. In the aquatic environment, moving towards prey generates a bow wave in front of the head, which will deviate the prey away from the mouth unless sufficient suction is generated (Lauder and Shaffer, 1985; Van Wassenbergh et al., 2010). A combination of blowing out water and the bow wave effect could push the prey away from the mudskipper when approaching the prey while the mouth is opening. Consequently, the mudskipper may have adapted its aquatic feeding system to function in the terrestrial environment to the detriment of its being able to feed under water. In other words, if the mudskipper is capable of feeding in both environments, there must be a considerable alteration in the functioning of its feeding system depending on its current environment.

In this study, we examine the function of the feeding apparatus of the mudskipper in the aquatic environment, and compare it to results described previously on its feeding in the terrestrial environment (Michel et al., 2015). This is not a direct comparative study of the functional morphology of the mudskipper's feeding system relative to that of other species of fish, but it is rather a comparative study of how the mudskipper manages to retain the functionally of its feeding system across two different environments. We will focus on the elements of the feeding system that are used in both environments, in particular the oral jaws, the hyoid, and the spatio-temporal volume changes of the head. Analysing the kinematics will allow us see how these elements are employed in each environment. This will answer the question whether aquatic feeding is compromised in the mudskipper, and whether and how environment-dependent adjustment of the feeding kinematics occurs. Ultimately, this will increase our understanding of how the conflicting biophysical demands of the aquatic and terrestrial environments can be dealt with by a vertebrate feeding system.

## RESULTS

**Fig. 1.**
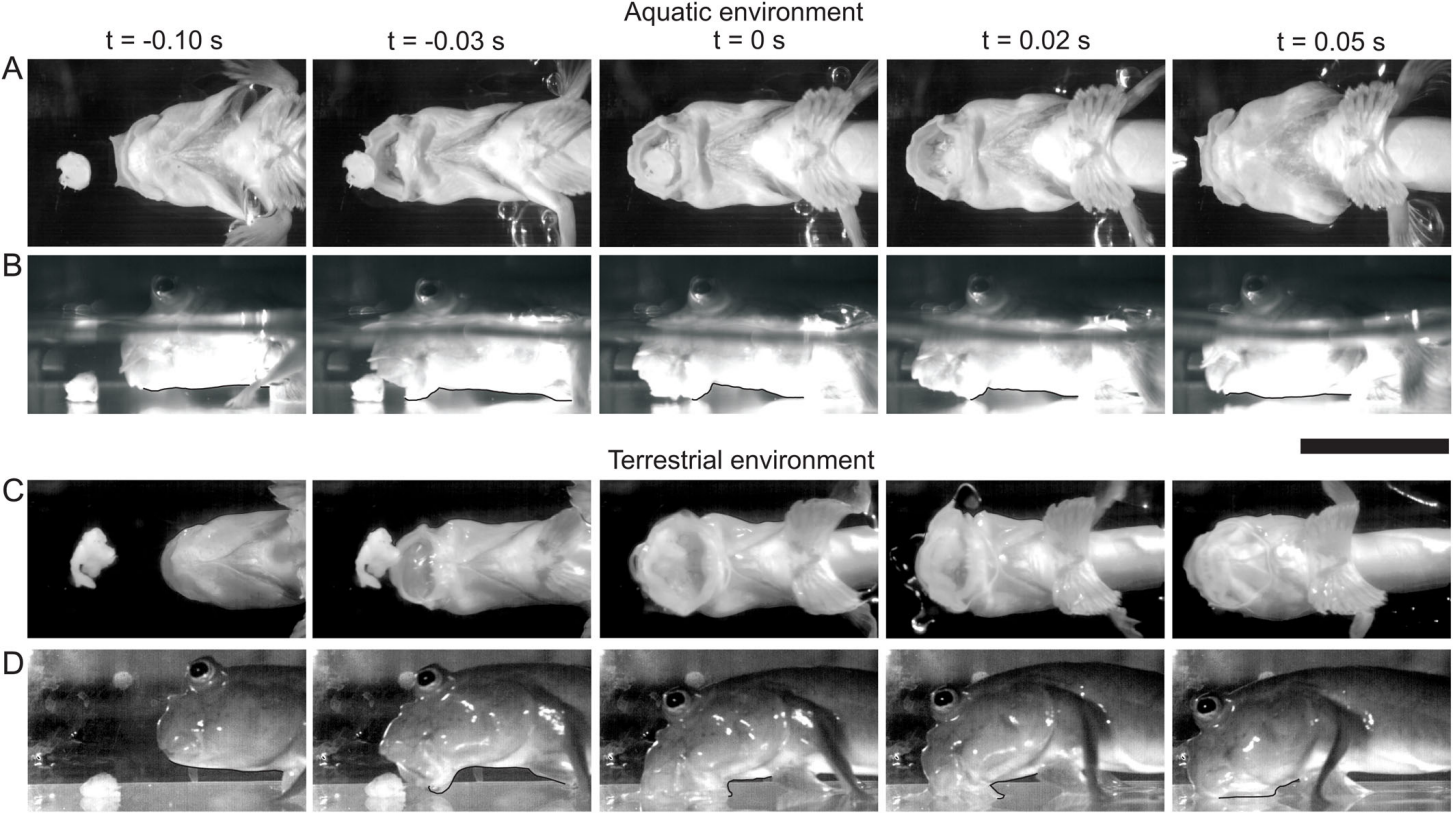
**High-speed video frames of feeding in *Periophthalmus barbarus*.** High-speed video frames showing successive stages of the prey capture event in both the aquatic environment (A,B) and the terrestrial environment (C,D). Both the lateral (A,C) and the ventral (B,D) views are shown. Images C and D were modified after Michel et al. (2015). Scale bar, 30 mm.

Here we present our data on aquatic feeding, and where relevant, we add the terrestrial data from Michel et al. (2015) for comparison. We will first describe the prey capture behaviour and associated movements of the Atlantic mudskipper in the aquatic environment. When under water, the mudskipper approaches its prey to a distance of approximately 2 cm before initiating its prey-capture movement (Fig. 1A,B). The mouth is opened as the mudskipper slowly approaches its prey. The gape of the mouth is increased and the head is expanded laterally at the height of the suspensorium and the opercula, during which time the prey item is moved towards and into the mouth. The mouth is then closed, which results in the prey item being either bitten into or captured in the buccal cavity (Fig. 1A,B; *t*=0.05). The gill slits are then opened and small particles in the water can be seen exiting from the gill openings as the opercula are adducted. Often a series of mouth openings and expansive movements are used to capture prey in the aquatic environment: when the water from the previous expansion movement has exited from the gills, the mouth is opened again and a new expansive movement is started by the mouth. The opercula are adducted while the mouth is opening for the following expansive movement, with the gill openings closed once the opercula are fully adducted (see Movie 1).

Terrestrial feeding is described *in extenso* by Michel et al. (2015). A brief summary follows: The mudskipper keeps its gill slits closed while on land, but when approaching its prey the opercula are adducted (Fig. 1C,D). Water often becomes visible in or around the mouth as the prey is being captured. Shortly after the maximum gape, the head is expanded, widening at the height of the suspensorium. This is followed by an abduction of the opercula (Fig. 1C,D; *t*=0.05). In some cases, the gill slits open after the prey is captured to release water (see Movie 2).

### Kinematics

**Fig. 2.**
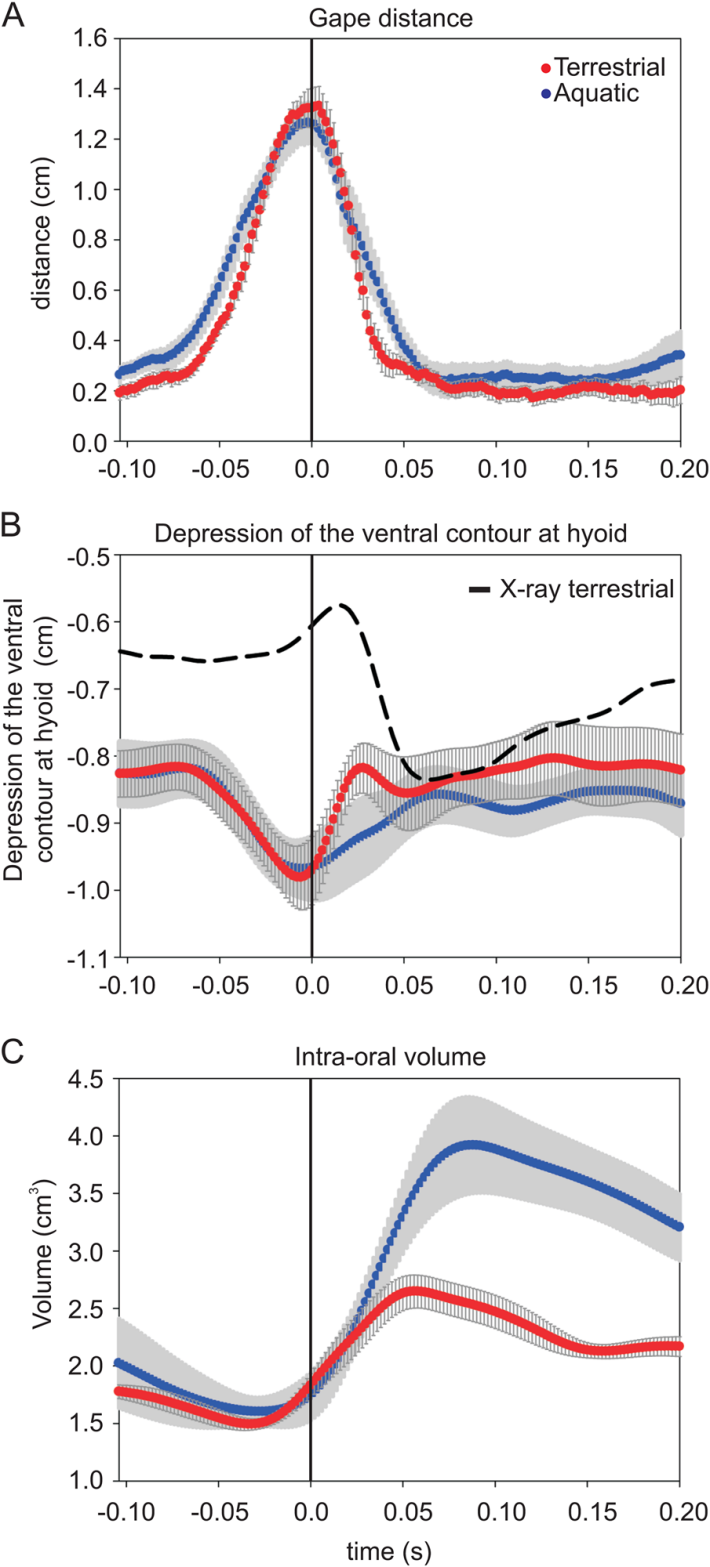
**Comparison of gape, hyoid and intra-oral volume during prey capture in *Periophthalmus barbarus*.** Mean kinematic profiles for aquatic (blue) and terrestrial (red) prey capture (*n*=4 individuals, *n*=8 feeding sequences per environment). *t*=0 was set as the moment of maximum gape. The kinematic profiles of gape distance (A), depression of the ventral contour of the head at the height of the hyoid (B), and total intra-oral volume (C) are shown. In B, the dotted line represents the hyoid depression and elevation measured by X-ray during terrestrial feeding from Michel et al. (2015). In C, the total average intra-oral volume change measured over the prey capture event is shown. Data are presented as means±s.e.m.

Kinematic profiles of mouth opening (gape distance), gill opening and hyoid depression were measured during the course of the feeding events (Fig. 2). Although there is no difference in the maximum gape distance between the aquatic and the terrestrial environments (*F*=0.479, *P*=0.25), there is a slight difference in the duration of the mouth opening. In the aquatic environment, the gape is open from *t*=−0.08±0.01 until 0.07±0.01, whereas in the terrestrial environment, the gape is open from *t*=−0.07±0.01 until 0.05±0.01 (*F*=4.832, *P*<0.04), where *t*=0 is the instant of maximum gape.

#### Gill opening

**Fig. 3.**
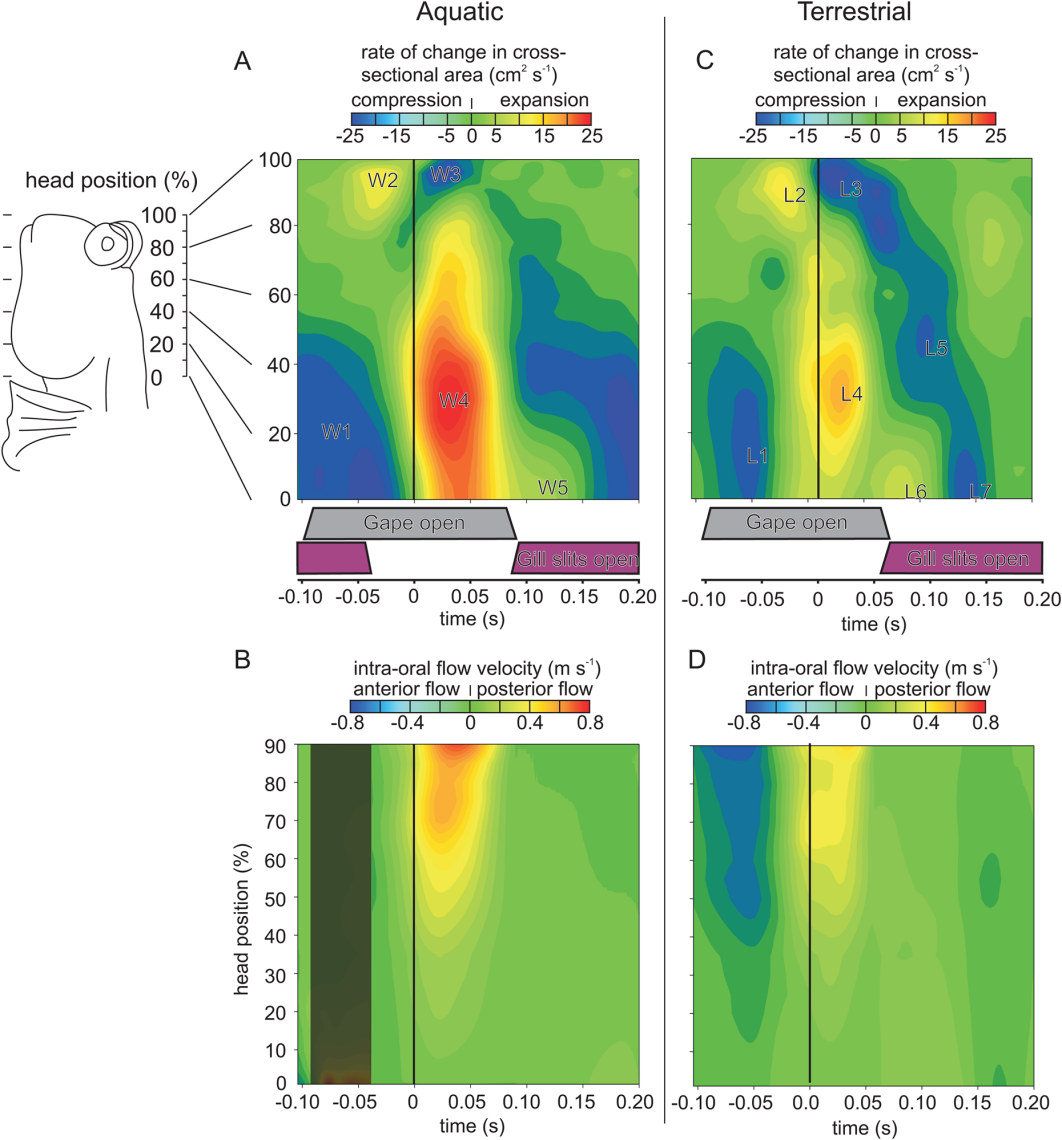
**Intra-oral rate of cross-sectional area change and flow velocity during prey capture in *Periophthalmus barbarus*.** The rate of change of the area of the oral cavity (A,C) and the flow velocities (B,D) along the length of the mudskipper's head during aquatic prey capture (A,B) and terrestrial prey capture (C,D; modified from data used and Fig. 2 in Michel et al., 2015). The vertical black lines on the graphs denotes *t*=0 (maximum mouth opening). The grey bars under graphs A and C illustrate the time during which the mouth is open, while the purple bars illustrate the time the gill slits are open. Values in A,C are spatio-temporally interpolated and averaged (two captures×four individuals) rates of change in the cross-sectional area, given as a function of the position along the head. This shows successive compression and expansion events: W1-W5 in A delineate zones of high compression or expansion during aquatic feeding, L1-L5 in C delineate zones of high compression or expansion during terrestrial feeding. In B,D the corresponding intra-oral flow velocities along the anterior-to-posterior axis are given, showing initially forwards (blue colouring) and then backwards motion (yellow to red colouring) of fluid in relation to the head. The dark area on B illustrates the time in which both gape and gills slits are open, and therefore flow velocity could not be calculated.

The mudskipper opercula are connected to the body by an opercular membrane which seals the ventral and almost the entire posterior end of the opercular cavity. The opening in the flexible membrane is a 1-cm-high slit. The timing of the opening and closing of the gill slits was measured in the ventral view of the high-speed video recordings. Unfortunately, because the gill slits were not visible from the lateral view, we were only able to get accurate data on the medial-lateral width of the opening. We therefore do not have any data on the complete area of the gill opening. We therefore considered any opening of the gills to allow full and unrestricted flow in or out of the gill slits. The time during which the gill slits were opened was measured over the feeding sequence for each individual in each environment (averages in purple bars in Fig. 3). In the aquatic environment, the gill slits were opened prior to the prey capture, and closed at *t*=−0.04±0.01. They opened again when the mouth was closed *t*=0.08±0.01. In the terrestrial environment, we found that the gill slits opened only when the mouth was closed *t*=0.06±0.01.

#### Hyoid

In the aquatic environment, the ventral contour of the head shows a depression at the level of the hyoid as the mouth opens (Fig. 2B). The maximum depression was around −0.85 cm just before the maximum mouth opening. In the terrestrial environment, the contour at the height of the hyoid started to elevate just after the maximum gape was reached. The moment of maximum elevation was around 0.03 s after the time of maximum gape.

### Volume changes and flow velocities

In the aquatic environment, the intra-oral volume is reduced prior to the maximum mouth opening (*t*=0) (Fig. 2C). As the maximum mouth opening is reached, the internal volume increases (*t*=−0.02±0.01) and continues, to reach a maximum intra-oral volume of around 4 cm^3^ (at *t*=0.09±0.01). However, in the terrestrial environment, the volume increase was initiated earlier (*F*=4.741, *P*=0.05) than in the aquatic environment (*t*=−0.03±0.01) and continues until a maximum buccal volume of 2.6 cm^3^ is reached (*t*=0.06±0.02).

In the aquatic environment, the rate of cross-sectional area change showed a reduction of volume in the posterior region of the head prior to the maximum gape (Fig. 3A, zone W1). The first spatio-temporal zone of expansion was at the snout and mouth opening (W2), which subsequently decreased after the maximum gape was reached (W3). Just before the maximum gape was reached, the zone posterior to 60% of the head length started to expand (W4). At *t*=0.03 the entire head posterior to 90% HL was expanded (W4). At *t*=0.08 the compression began, with the exception of the very posterior zone, around 5% HL (W5).

The intra-oral flow velocity in the aquatic environment could not be calculated between *t*=−0.09 and *t*=−0.04, since during this time both the mouth and gill slits were open (Fig. 3B). After *t*=−0.04, only the mouth remained open and the intra-oral flow could again be calculated. From around *t*=−0.03 a flow in the posterior direction started to be generated, reaching peak flow velocity after the maximum mouth opening. The flow velocity diminished towards the posterior of the head when the mouth was closing. From the instant the mouth was closed, around *t*=0.07, the gill slits were again opened, allowing a low-velocity, posterior-directed water flow to exit the head.

## DISCUSSION AND CONCLUSIONS

In order to draw a comparison with the data from the aquatic environment, a qualitative description of the pattern observed in the terrestrial environment is provided. This is based on data from Michel et al. (2015). To make such a comparison possible, in Fig. 3C,D the terrestrial data are presented on the same scale as the aquatic data. On land, prior to the maximum mouth opening, there was a slight reduction of the total volume of the head (Fig. 2C). This reduction in volume was due to the compression of the posterior zone of the mudskipper's head (Fig. 3C, zone L1). With the mouth open and the gill slits closed (Fig. 1A), a flow toward the mouth was generated (Fig. 3D). At around *t*=−0.02, nearing the time of maximum gape, the total volume of the head started to increase (Fig. 2C). At the very anterior end of the head, the opening of the mouth rapidly increased the cross-sectional area of the mouth (Fig. 3C, zone L2). The cross-sectional areas posterior to 80% HL then started to expand (Fig. 3C, zone L4), reversing the anterior flow to a flow that was posterior in direction (Fig. 3D). In the terrestrial environment, the total volume of the head continued to increase as the maximum gape was reached (Fig. 2C). The closing of the mouth then rapidly decreased the cross-sectional area of the anterior end of the mouth (Fig. 3C, zone L3). Starting from the anterior end of the buccal cavity, we identified three zones of compression (Fig. 3C, zones L3, L5 and L7), but in each case the posterior zones continued to expand (Fig. 3C, zones L4 and L6). This allowed for a further posterior intra-oral flow (Fig. 3D). At around *t*=0.05, the mouth was closed and the gill slits started to open. The total intra-oral volume now decreased with a wave of compression that started at the anterior end and finally reached the posterior end of the head (Fig. 3C, zone L7). This created a low-velocity flow directed towards the gill slits (Fig. 3D).

When we compare the mudskipper's kinematics in the aquatic and the terrestrial environments, we find that the magnitude and timing of the compression and expansion movements of the head differ according to the environment. The compression and expansion movements of the head are reduced in the terrestrial environment in comparison to those in the aquatic environment. In addition, we found that the onset of the overall volume increase and the posterior intra-oral flow started slightly earlier in the terrestrial environment relative to that in the aquatic environment (Fig. 2C; Fig. 3B,D). This is similar to the pattern found in the more amphibious salamanders and turtles, where there is a reduction in the size of movements in response to the terrestrial environment. In a previous study on turtles, naïve aquatic individuals would employ longer and more extensive movements of the gape and hyoid in order to feed on land (Stayton, 2011). This is the most likely response when essentially the same motor patterns are used in water as on land (Stayton, 2011). The movements measured for the eel-catfish (*Channallabes apus*) when feeding in the terrestrial environment were similar in that the hyoid and jaw depression were larger, but the duration of the movements was shorter than those in the aquatic environment (Van Wassenbergh, 2013). The more extensive movements on land might be the result of similar levels of activation of the muscles with a lack of the fluid dynamic resistance which would be present in the aquatic environment (Stayton, 2011). In the more terrestrially inclined species of the amphibious turtles and salamanders, the movements of the hyoid are reduced in response to feeding in terrestrial environments (Shaffer and Lauder, 1985; Summers et al., 1998; Stayton, 2011). In our study, we found that the mudskipper, similarly, was capable of larger compressive and expansive movements in the aquatic environment, but that most of these movements were reduced in response to the terrestrial environment.

The depression of the ventral contour at the level of the hyoid is different in the aquatic and the terrestrial environments. It should be noted that the ventral contour of the head at the height of the hyoid is influenced by the opening of the lower jaw. During prey capture in the terrestrial environment, the lower jaw is depressed and rotated over 90°, this positions the lower jaw close to the height of the hyoid (Michel et al., 2015). Despite these obstructions, we were able to measure a difference in depression at the hyoid height in the aquatic and terrestrial environments (Fig. 2B). In the aquatic environment, the depression of the hyoid followed the opening of the mouth as part of the expansion of the buccal cavity. This pattern of hyoid depression during the opening of the jaws is similar to that found in other vertebrates feeding in the aquatic environment (Summers et al., 1998; Heiss et al., 2013). On land, the elevation of the ventral contour at the level of the hyoid is observed just after the mouth opens (Fig. 2B). If we compare the terrestrial ventral contour at the level of the hyoid with the movement shown by the X-ray data of the hyoid from Michel et al. (2015), we find that they are not the same (Fig. 2B, terrestrial X-ray curve). This is probably due to our inability to follow the tip of the hyoid accurately from the lateral view. The depression of the ventral contour of the hyoid in the terrestrial environment is likely mostly due to the depression of the lower jaw, of which the proximal end obscures the distal tip of the hyoid in lateral view. The elevation which follows shortly after the depression is probably the contour returning to the ventral outline of the hyoid, which is depressed after the maximum mouth opening (see X-ray curve in Fig. 2B). In amphibious tetrapods feeding terrestrially, the hyoid is either elevated during maximum mouth opening and thus not used, or it is slightly depressed after maximum gape (Summers et al., 1998; Heiss et al., 2013). Here we see evidence of a slight depression in the mudskipper after maximum gape in terrestrial prey capture. Although the depression of the lower jaw may also have affected the aquatic hyoid contour, the lack of elevation after maximum mouth opening indicates that the hyoid is still depressed in the aquatic environment. This suggests that the mudskipper changes the depression of the hyoid in response to the feeding environment.

The final difference between prey capture in the aquatic and terrestrial environments is the intra-oral flow which results from the expansive and compressive movements of the mudskipper's head. In the aquatic environment, we found a relatively typical suction feeding sequence. In preparation for capturing prey, water is evacuated from the opercular cavity via the gill slits by compression, followed by expansive movements of the head, which draw in a water flow through the mouth (Fig. 1A,B; Fig. 3; Movie 1). During terrestrial feeding, we find a similar pattern of expansive and compressive movements in the mudskipper's head, but they are less extensive. However, these now result in an anterior flow of water toward the mouth opening (Fig. 3C,D) (Michel et al., 2015). This is followed by a posterior flow, in which water is expelled from the gill slits after prey capture (Fig. 1C,D; Fig. 3D; Movie 1). In both environments, the pattern of compressive and expansive movements of the head are similar prior to maximum mouth opening (Fig. 3A,C). In both, the posterior of the head is compressed, while the anterior is expanding. The difference in flow comes from the timing of the gill slit closure: when closed, an anterior flow is generated; when opened, water can exit the head posteriorly.

Based on the timing of the gill slit opening, we can conclude that the mudskipper is capable of controlling this opening. Without the membranes that enclose the gill chambers, the mudskipper would not be able to generate suction through the mouth in the aquatic environment, and the abduction of the opercula would cause water to flow into the gill chambers through the gill slits. Similarly, the intra-oral water in the terrestrial environment could not be directed fully in the anterior direction and would probably at least partly flow out through the gill slits. Relative to the size of its opercula, the mudskipper has large adductor operculi and levator operculi muscles at some distance from the joint between the opercula and the hyomandibular (see Fig. 2 in Michel et al., 2014). These muscles are possibly used to help seal the gill chambers. Alternatively, the adductor hyohyoideus muscles possibly assist in closing the gill slits independently of the opercular musculature (Spierts et al., 2003). However, we cannot be sure whether these muscles are responsible for controlling the opening of the gill slits. The gills slits possibly act as a valve system, using the partial pressure differences to ‘passively’ seal the gill chambers. However, based on the kinematics of the opercula and observations of the gill slit opening, we can see modification of the pattern in response to the prevailing environment.

By studying the prey capture method of the mudskipper in the aquatic environment and then comparing it with its terrestrial prey capture method, we found a very clear modification in the way the feeding apparatus is used in these two environments. The mudskipper is capable of benthic suction feeding in the aquatic environment. It uses rapid and extensive expansion of its buccal cavity to generate a flow of water from the external environment into the mouth in a manner very similar to that of fully aquatic fish. We found evidence that the hyoid may be used to aid in the expansion of the buccal cavity in a manner similar to that of a fully aquatic fish. This is contrasted with its use of its feeding system in the terrestrial environment, where there is a slightly earlier but reduced compressive and expansive movement of the elements along the head; the sealed gill slits and a considerable elevation of the hyoid aid in capturing prey when on land. We see a similar trend towards a reduction in the expansive movements of the head and hyoid in the more terrestrially inclined amphibious tetrapods when feeding underwater compared to on land (Summers et al., 1998; Stayton, 2011; Heiss et al., 2013). Although this does not apply to gape, we find that the mudskipper modulates the use of the elements of its feeding apparatus in response to its medium in a manner that is similar to that of the amphibious tetrapods. In this way, the feeding system of the mudskipper can function in both environments as it uses a single set of anatomical structures across the two disparate environments.

As previously stated in Michel et al. (2015), the tetrapodomorphs and the modern sarcopterygians clearly differ in morphology from the mudskippers, but the main functional elements of the mudskipper's feeding system are also present in those groups. A similar usage of the feeding system to capture prey at some stage during the early evolution of the tetrapods' lineage can therefore not be excluded on morphological grounds. The similarities in the motion pattern of the hyoid between mudskippers and the tongue-protruding salamanders have lead us to question the current hypothesis about the evolution of terrestrial feeding behaviour in the early tetrapods (Michel et al., 2015). We propose that a hydrodynamic tongue, such as we find in the mudskippers when feeding on land, may have evolved to move the prey grabbed between the jaws. In this study, we found that the elevation followed by depression of the floor of the mouth by means of the hyoid skeleton as exhibited on land, is retained from a behaviour in which intra-oral water is used in the aquatic environment. An already established kinematic pattern of aquatic suction feeding could have allowed for water-mediated terrestrial feeding by means of gradual anatomical specialization towards better terrestrial capture of prey. This would have been achieved by better control of the opening of the gill slits and of the intra-oral volume changes. This further supports our hypothesis that water-mediated terrestrial feeding may have been an important intermediate step in the colonization of land, as the transition from aquatic feeding to the terrestrial usage of a hydrodynamic tongue only required a small modulation in cranial kinematics while aquatic feeding still remained possible.

## MATERIALS AND METHODS

### Study species

Five adult individuals of Atlantic mudskippers *Periophthalmus barbarus* (Linnaeus, 1766) were obtained through the commercial pet trade. The standard length (measured from the snout tip to the posterior end of the last vertebra) of the five individuals was similar (9.9±1.8 cm). One individual was euthanised by using an overdose of MS-222 (Sigma Chemical) and used for computed tomography (CT) scanning (the scanning protocol was described previously by Michel et al., 2014). The mudskippers were kept in a large aquarium (200 l) at a constant temperature of 27°C with a 12L:12D photoperiod cycle. For filming sessions, the animals were transferred to a small plexiglass aquarium (30 liters). The mudskippers were trained to capture prey in a narrow corridor extending along one side of the aquarium to increase the chances of filming the animals in a position perpendicular to the camera. Food was always presented on the bottom of the tanks. The water level was always above the gill slits. All of the specimens used in this study were handled according to University of Antwerp Animal Care protocols.

### High-speed video recordings

Two high-speed cameras were used to record the movements of the feeding apparatus in the lateral and ventral plane simultaneously: a Redlake Motionscope M3 and a Redlake MotionPro HS1000 (Redlake Inc., Tallahassee, FL, USA) with a recording speed of 500 fps (1280×1024 pixels). Several bright LEDs provided the necessary illumination. Of all the lateral view recordings, only those with the lateral side of the head orientated sufficiently perpendicular to the camera lens axis were retained for further analysis. For each individual, two successful feeding events were analysed. The instant of maximum gape was set as *t*=0 s.

### Volume and flow velocities

**Fig. 4.**
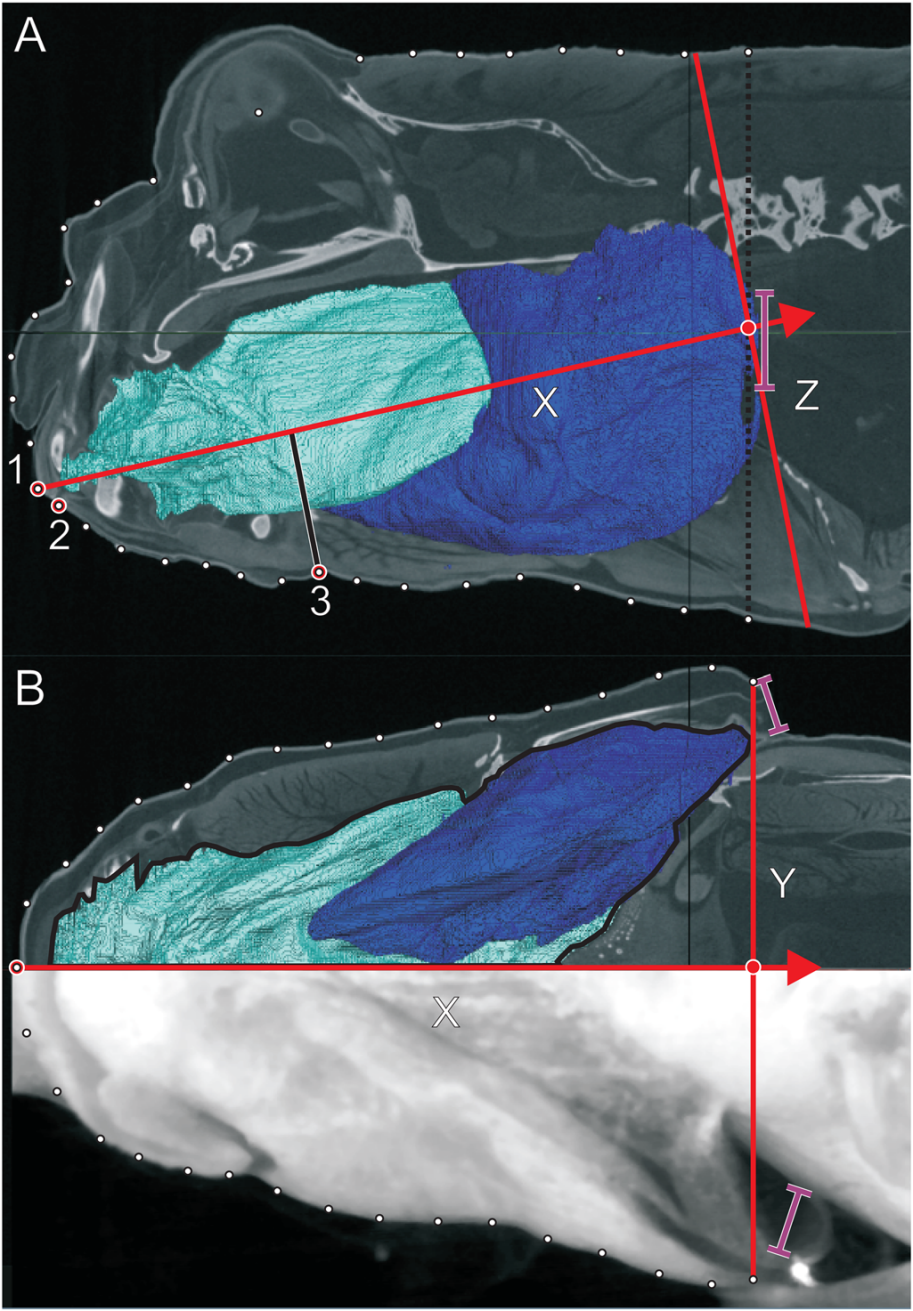
**CT scan of *Periophthalmus barbarus* showing the intra-oral volume relative to the outer contour.** CT scan cross-section illustrating the outer contours of the head in relation to the oral volume in front of the gills (cyan) and behind the gills (blue). The red lines illustrate the fish-bound axes of measurement for each frame; the red arrow indicates the direction of positive flow velocities. (A) Lateral view with contour landmarks (white points) and the fish-bound axes (red lines) from mid of the opercula (dotted black line) to the upper jaw tip (1). Additional kinematic landmarks are the lower jaw tip (2) and the point of the hyoid along the ventral outline of the body (3), measured from the midline (solid black line). (B) Ventral view with the contour landmarks, the buccal-pharyngeal cavity and the fish-bound axes (red lines). The purple lines denote the position of open gill slits.

The ellipse method of measuring the volume of biological objects was first proposed and applied by Drost and van den Boogaart (1986). They established the use of this method for a wide range of biologically relevant applications. In addition, they tested and validated their model for flow velocities in a suction feeding fish (Drost and van den Boogaart, 1986). Further validation of the model was performed by Aerts et al. (2001) on suction feeding in turtles. Using the ellipse method, the mudskipper head is modelled as 21 elliptical cylinders of which the axes of the ellipse surface correspond to the width and height of the head. The height of each cylinder is defined as the length of the head divided by 21 (Fig. 4).

The elliptical cylinders were set based on a fish-bound frame of reference for each frame of each video. In the lateral view, the X-axis was defined as the line connecting the tip of the upper jaw and the middle of the body at the operculum (Fig. 4A). The middle of the opercula was set as the origin of the fish-bound frame of reference. In the dorsal view, the X-axis was determined as the line connecting the snout tip to the middle point between the opercula (Fig. 4B). The contours of the head were digitised frame by frame for each video using 30 landmarks for each frame, 15 on each side of the head for the ventral and lateral views using ImageJ (NIH, TX, USA). The contours of the head were then divided into 21 evenly spaced distances using linear interpolation, perpendicular to the respective X-axes (Fig. 4). This gave us 21 heights and widths for each video frame. In order to reduce data noise caused by manual digitisation, a fourth-order lowpass, zero phase-shift Butterworth filter was used, with a cut-off frequency of 15 Hz.

The initial internal volume of the bucco-pharyngeal cavity was obtained by the aforementioned CT-scan of a mudskipper. To visualize the dimensions, the bucco-pharyngeal cavity was sectioned and colour coded using Amira (Mercury Systems). This allowed us to accurately determine the boundaries of the bucco-pharyngeal cavity relative to the outer contours (Fig. 4). As with the frames of the video recordings, the outer contours of the animal and the boundaries of the bucco-pharygeal cavity in the CT-scan were divided into 21 evenly spaced distances using linear interpolation. In this way, the volume of each cylinder was calculated, providing the volume of the buccal cavity before the start of the prey-capture event.

We assumed that the volume of the tissues of the head remained constant, therefore any volume change, based on the outer contours of the head, must equal a change of volume of the bucco-pharyngeal cavity. The law of continuity dictates that any volume increase of the bucco-pharyngeal cavity must immediately be filled with water. Therefore, any volume change would create a flow to, or from the cavity. Because we knew the cross-sectional area at different lengths along the head, we could calculate the instantaneous flow velocity in these areas, based on the change in volume behind it. This calculation of the flow velocity is only possible if either the mouth or the opercular slits are open. If both are open, it is unclear how the volumetric change affects the direction or magnitude of the flow velocity along the bucco-pharyngeal cavity. In each high-speed video, the instant of the mouth opening was determined. The mouth was considered open until the jaws were closed after prey capture. The same was assumed for the opercular opening: once opened, they were assumed open until the gill covers were fully adducted. Fluid flow was calculated along the X-axis of the fish-bound frame of reference, along the line of the upper jaw to the opercula. It was assumed that prey items behave as water particles in these calculations.

### Kinematic variables

In the video recordings of the lateral view, the following landmark coordinates were determined for each frame in addition to the contours: (1) tip of the upper jaw, (2) tip of the lower jaw, (3) the point along the ventral outline of the head at the height of the hyoid (see Fig. 4). Using these land marks, two kinematic variables were measured for the duration of each prey-capture sequence: gape distance and hyoid depression relative to the fish-bound axis. Any hyoid elevation (i.e. when the hyoid tip was lying between the suspensoria) was obscured by the suspensorium and could therefore not be measured.

### Comparison with terrestrial feeding kinematics

In this study, data from the terrestrial feeding in *Periophthalmus barbarous,* as described by Michel et al. (2015), were used for comparison with the aquatic data. However, because no X-ray recordings could be used in the aquatic environment, only data from external video recordings in the terrestrial environment were used. To do this, the ventral outline of the head at the height of the hyoid was used in both environments. This comparison required a recalculation of the terrestrial kinematics (i.e. it no longer used the correction factor for hyoid elevation as described in Michel et al., 2015). Videos used in Michel et al. (2015) are available via Dryad at http://dx.doi.org/10.5061/dryad.0fg55.
